# DualFlow: Generating imperceptible adversarial examples by flow field and normalize flow-based model

**DOI:** 10.3389/fnbot.2023.1129720

**Published:** 2023-02-09

**Authors:** Renyang Liu, Xin Jin, Dongting Hu, Jinhong Zhang, Yuanyu Wang, Jin Zhang, Wei Zhou

**Affiliations:** ^1^School of Information Science and Engineering, Yunnan University, Kunming, China; ^2^Engineering Research Center of Cyberspace, Yunnan University, Kunming, China; ^3^National Pilot School of Software, Yunnan University, Kunming, China; ^4^School of Mathematics and Statistics, University of Melbourne, Melbourne, VIC, Australia; ^5^Kunming Institute of Physics, Yunnan University, Kunming, China

**Keywords:** deep learning, adversarial attack, adversarial example, normalize flow, spatial transform

## Abstract

Recent adversarial attack research reveals the vulnerability of learning-based deep learning models (DNN) against well-designed perturbations. However, most existing attack methods have inherent limitations in image quality as they rely on a relatively loose noise budget, i.e., limit the perturbations by *L*_*p*_-norm. Resulting that the perturbations generated by these methods can be easily detected by defense mechanisms and are easily perceptible to the human visual system (HVS). To circumvent the former problem, we propose a novel framework, called **DualFlow**, to craft adversarial examples by disturbing the image's latent representations with spatial transform techniques. In this way, we are able to fool classifiers with human imperceptible adversarial examples and step forward in exploring the existing DNN's fragility. For imperceptibility, we introduce the flow-based model and spatial transform strategy to ensure the calculated adversarial examples are perceptually distinguishable from the original clean images. Extensive experiments on three computer vision benchmark datasets (CIFAR-10, CIFAR-100 and ImageNet) indicate that our method can yield superior attack performance in most situations. Additionally, the visualization results and quantitative performance (in terms of six different metrics) show that the proposed method can generate more imperceptible adversarial examples than the existing imperceptible attack methods.

## 1. Introduction

Deep neural networks (DNNs) have achieved remarkable achievements in theories and applications. However, the DNNs have been proven to be easily fooled by adversarial examples (AEs), which are generated by adding well-designed unwanted perturbations to the original clean data (Zhou et al., 2019). In these years, many studies dabbled in crafting adversarial examples and revealed that many DNN applications are vulnerable to them. Such as Computer Vision (CV) (Kurakin et al., 2017; Eykholt et al., 2018; Duan et al., 2020), Neural Language Processing (NLP) (Xu H. et al., 2020; Shao et al., 2022; Yi et al., 2022), and Autonomous Driving (Liu A. et al., 2019; Zhao et al., 2019; Yan et al., 2022). Generally, in CV, the AE needs to meet the following two properties, one is that it can attack the target model successfully, resulting in the target model outputting wrong predictions; another one is its perturbations should be invisible to human eyes (Goodfellow et al., 2015; Carlini and Wagner, 2017).

Unfortunately, most existing works (Kurakin et al., 2017; Dong et al., 2018, 2019) are focused on promoting the generated adversarial examples' attack ability but ignored the visual aspects of the crafted evil examples. Typically, the calculated adversarial noise is limited by a small *L*_*p*_-norm ball, which tries to keep the built adversarial examples looking like the original image as possible. However, the *L*_*p*_-norm limited adversarial perturbations blur the images to a large extent and are so conspicuous to human eyes and not harmonious with the whole image. Furthermore, these *L*_*p*_-norm-based methods, which modify the entire image at the pixel level, seriously affect the quality of the generated adversarial images. Resulting in the vivid details of the original image can not be preserved. Besides, the adversarial examples crafted in these settings can be easily detected by the defense mechanism or immediately discarded by the target model and further encounter the “denied to service.” All the mentioned above can lead the attack to be failed. Furthermore, most existing methods adopt *L*_*p*_-norm, i.e., *L*_2_ and *L*_*inf*_-norm, distance as the metrics to constraint the image's distortion. Indeed, the *L*_*p*_-norm can ensure the similarity between the clean and adversarial images. However, it does not perform well in evaluating an adversarial example.

Recently, some studies have attempted to generate adversarial examples beyond the *L*_*p*_-norm ball limited way. For instance, patch-based adversarial attacks, which usually extend into the physical world, do not limit the intensity of perturbation but the range scope. Such as adversarial-Yolo (Thys et al., 2019), DPatch (Liu X. et al., 2019), AdvCam (Duan et al., 2020), Sparse-RS (Croce et al., 2022). To obtain more human harmonious adversarial examples with acceptable attack success rate in the digital world, Xiao et al. (2018) proposed the stAdv to generate adversarial examples by spatial transform to modify each pixel's position in the whole image. The overall visual effect of the adversarial example generated by stAdv is good. However, the adversarial examples generated by stAdv usually have serration modifications and are visible to the naked eye. Later, the Chroma-Shift (Aydin et al., 2021) made a forward step by applying the spatial transform to the image's YUV space rather than RGB space. Unfortunately, these attacks have destroyed the semantic information and data distribution of the image, resulting that the generated adversarial noise that can be easily detected by the defense mechanism (Arvinte et al., 2020; Xu Z. et al., 2020; Besnier et al., 2021) and leading the attack failed.

To gap this bridge, we formulate the issue of synthesizing invisible adversarial examples beyond noise-adding at pixel level and propose a novel attack method called **DualFlow**. More specifically, DualFlow uses spatial transform techniques to disturb the latent representation of the image rather than directly adding well-designed noise to the benign image, which can significantly improve the adversarial noise's concealment and preserve the adversarial examples' vivid details at the same time. The spatial transform can learn a smooth flow field vector *f* for each value's new location in the latent space to optimize an eligible adversarial example. Furthermore, the adversarial examples are not limited to *L*_*p*_-norm rules, which can guarantee the image quality and details of the generated examples. Empirically, the proposed DualFlow can remarkably preserve the images' vivid details while achieving an admirable attack success rate.

**Figure 1:**
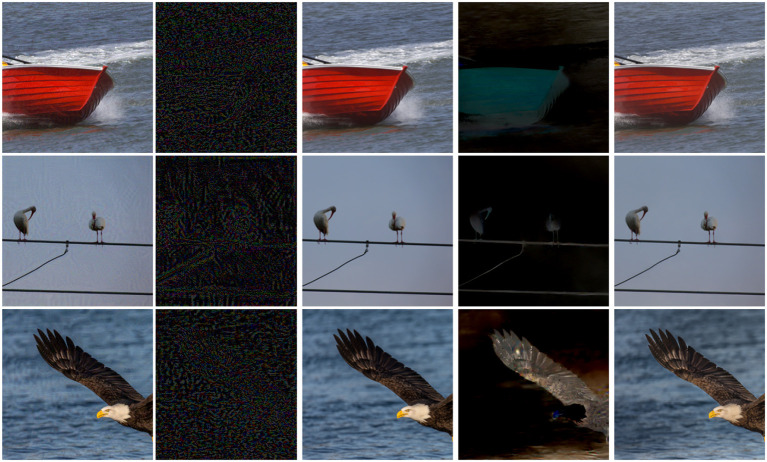
The adversarial examples generated by the MI-FGSM (Aydin et al., 2021) and the proposed DualFlow for the ResNet-152 (He et al., 2016) model. Specifically, the first column and the second column are the adversarial examples and their corresponding adversarial perturbations generated by MI-FGSM, respectively. The middle column is the clean images. The last two columns are the adversarial perturbations and their corresponding adversarial examples, respectively.

We conduct extensive experiments on three different computer vision benchmark datasets. Results illustrate that the adversarial perturbations generated by the proposed method take into account the data structure and only appear around the target object. We draw the adversarial examples and their corresponding noise from the noise-adding method MI-FGSM and the DualFlow in Figure 1. As shown in Figure 1, our proposed method slightly alters this area around the target object, thus ensuring the invisibility of the adversarial perturbations. Furthermore, the statistical results demonstrate that the DualFlow can guarantee the generated adversarial examples' image quality compared to the existing imperceptible attack methods on the target models while outperforming them both on the ordinary and defense models concerning attack success rate. The main contributions could be summarized as follows:

- We propose a novel attack method, named DualFlow, which generates adversarial examples by directly disturbing the latent representation of the clean examples rather than performing an attack on the pixel level.
- We craft the adversarial examples by applying the spatial transform techniques to the latent value to preserve the details of original images and guarantee the adversarial images' quality.
- Comparing with the existing attack methods, experimental results show our method's superiority in synthesizing adversarial examples with the highest attack ability, best invisibility, and remarkable image quality.

The rest of this paper is organized as follows. First, we briefly review the methods relating to adversarial attacks and imperceptible adversarial attacks in Section 2. Then, Sections 3 and 4, introduce the preliminary knowledge and the details of the proposed DualFlow framework. Finally, the experimental results are presented in Section 5, with the conclusion drawn in Section 6.

## 2. Related work

In this section, we briefly review the most pertinent attack methods to the proposed work: the adversarial attacks and the techniques used for crafting inconspicuous adversarial perturbations.

### 2.1. Adversarial attack

Previous researchers contend that deep neural networks (DNN) are sensitive to adversarial examples (Goodfellow et al., 2015), which are crafted by disturbing the clean data slightly but can fool the well-trained DNN models. The classical adversarial attack methods can be classified into two categories, white-box attacks (Kurakin et al., 2017; Madry et al., 2018) and black-box attacks (Narodytska and Kasiviswanathan, 2017; Bai et al., 2023). In white-box settings, the attackers can generate adversarial examples with a nearly 100% attack success rate because they can access the complete information of the target DNN model, while for the physical world, the black-box attack is more threatening to the DNN applications because they don't need too much information about the DNN models' details (Ilyas et al., 2018, 2019; Guo et al., 2019).

### 2.2. Imperceptible adversarial attacks

Recently, some studies have attempted to generate adversarial examples beyond the *L*_*p*_-norm ball limit for obtaining humanly imperceptible adversarial examples. LowProFool (Ballet et al., 2019) propose an imperceptibility attack to craft invisible adversarial examples in the tabular domain. Its empirical results show that LowProFool can generate imperceptible adversarial examples while keeping a high fooling rate. For computer vision tasks the attackers will also consider the human perception of the generated adversarial examples. In Luo et al. (2018), the authors propose a new approach to craft adversarial examples, which design a new distance metric that considers the human perceptual system and maximizes the noise tolerance of the generated adversarial examples. This metric evaluates the sensitivity of image pixels to the human eye and can ensure that the crafted adversarial examples are highly imperceptible and robust to the physical world. stAdv (Xiao et al., 2018) focuses on generating different adversarial perturbations through spatial transform and claims that such adversarial examples are perceptually realistic and more challenging to defend against with existing defense systems. Later, the Chroma-Shift (Aydin et al., 2021) made a forward step by applying the spatial transform to the image's YUV space rather than RGB space. AdvCam (Duan et al., 2020) crafts and disguises adversarial examples of the physical world into natural styles to make them appear legitimate to a human observer. It transfers large adversarial perturbations into a custom style and then “hides” them in a background other than the target object. Moreover, its experimental results that AEs produced by AdvCam are well camouflaged and highly concealed in both digital and physical world scenarios while still being effective in deceiving state-of-the-art DNN image detectors. SSAH (Luo et al., 2022) crafts adversarial examples and disguises adversarial noise in a low-frequency constraints manner. This method limits the adversarial perturbations to the high-frequency components of the specific image to ensure low human perceptual similarity. The SSAH also jumps out of the original *L*_*p*_-norm constraint-based attack way and provides a new idea for calculating adversarial noise.

Therefore, crafting adversarial examples, especially for the imperceptible ones, poses the request for a method that can efficiently and effectively build adversarial examples with high invisibility and image quality efficiently and effectively. On the other hand, with the development of defense mechanisms, higher requirements are placed on the defense resistance of adversarial examples. To achieve these goals, we learn from the previous studies that adversarial examples can be gained beyond noise-adding ways. Hence, we are well motivated to develop a novel method to disturb the original image latent representation obtained by a well-trained normalizing flow-based model, and then apply a well-calculated flow field to it to generate adversarial examples. Our method can build adversarial examples with high invisibility and image quality without losing attack performance.

## 3. Preliminary

Before introducing the details of the proposed framework, in this section, we first present the preliminary knowledge about adversarial attacks and normalizing flows.

### 3.1. Adversarial attack

Given a well-trained DNN classifier C and a correctly classified input (*x, y*)~*D*, we have C(x)=y, where *D* denotes the accessible dataset. The adversarial example *x*_*adv*_ is a neighbor of *x* and satisfies that $C\left(x_{adv}\right)\neq y$ and ||*x*_*adv*_−*x*||_*p*_ ≤ ϵ, where the ℓ_*p*_ norm is used as the metric function and ϵ is usually a small value such as 8 and 16 with the image intensity [0, 255]. With this definition, the problem of calculating an adversarial example becomes a constrained optimization problem:


(1) $$x_{adv}=\underset{{\left\|x_{adv}-x\right\|}_{p}\leq \epsilon }{arg\;max\;\ell }(C(x_{adv})\neq y),$$

Where ℓ stands for a loss function that measures the confidence of the model outputs.

In the optimization-based methods, the above problem is solved by computing the gradients of the loss function in Equation (1) to generate the adversarial example. Furthermore, most traditional attack methods craft adversarial examples by optimizing a noise δ and adding it to the clean image, i.e., *x*_*adv*_ = *x*+δ. By contrast, in this work, we formulate the *x*_*adv*_ by disturbing the image's latent representation with spatial transform techniques.

### 3.2. Normalizing flow

The normalizing flows (Dinh et al., 2015; Kingma and Dhariwal, 2018; Xu H. et al., 2020) are a class of probabilistic generative models, which are constructed based on a series of entirely reversible components. The reversible property allows to transform from the original distribution to a new one and vice versa. By optimizing the model, a simple distribution (such as the Gaussian distribution) can be transformed into a complex distribution of real data. The training process of normalizing flows is indeed an explicit likelihood maximization. Considering that the model is expressed by a fully invertible and differentiable function that transfers a random vector *z* from the Gaussian distribution to another vector *x*, we can employ such a model to generate high dimensional and complex data.

⊮ Specifically, given a reversible function *F*:ℝ^*d*^ → ℝ^*d*^ and two random variables *z*~*p*(*z*) and *z*′~*p*(*z*′) where *z*′ = *f*(*z*), the change of variable rule tells that


(2) $$p(z^{\prime })=p(z)\left|det\frac{\partial {\text{F}}^{-1}}{\partial z^{\prime }}\right|,$$

(3) $$p(z)=p(z^{\prime })\left|det\frac{\partial \text{F}}{\partial z}\right|,$$

Where *det* denotes the determinant operation. The above equation follows a chaining rule, in which a series of invertible mappings can be chained to approximate a sufficiently complex distribution, i.e.,


(4) $$\begin{array}{l} z_{K}={\text{F}}_{K}⊙\ldots ⊙{\text{F}}_{2}⊙{\text{F}}_{1}\left(z_{0}\right), \end{array}$$

Where each *F* is a reversible function called a flow step. Equation (4) is the shorthand of *F*_*K*_(*F*_*k*−1_(…*F*_1_(*x*))). Assuming that *x* is the observed example and *z* is the hidden representation, we write the generative process as


(5) $$\begin{array}{l} x={\text{F}}_{\theta }\left(z\right), \end{array}$$

Where *F*_θ_ is the accumulate sum of all *F* in Equation (4). Based on the change-of-variables theorem, we write the log-density function of *x* = *z*_*K*_ as follows:


(6) $$-\log p_{K}(z_{K})=-\log p_{0}(z_{0})-\sum_{k=1}^{K}\log\left|det\frac{\partial z_{k-1}}{\partial z_{k}}\right|,$$

Where we use *z*_*k*_ = *F*_*k*_(*z*_*k*−1_) implicitly. The training process of normalizing flow is minimizing the above function, which exactly maximizes the likelihood of the observed training data. Hence, the optimization is stable and easy to implement.

### 3.3. Spatial transform

The concept of spatial transform is firstly mentioned in Fawzi and Frossard (2015), which indicates that the conventional neural networks are not robust to rotation, translation and dilation. Next, Xiao et al. (2018) utilized the spatial transform techniques and proposed the stAdv to craft adversarial examples with a high fooling rate and perceptually realistic beyond noise-adding way. StAdv changes each pixel position in the clean image by applying a well-optimized flow field matrix to the original image. Later, Zhang et al. (2020) proposed a new method to produce the universal adversarial examples by combining the spatial transform and pixel distortion, and it successfully increased the attack success rate against universal perturbation to more than 90%. In the literature (Aydin et al., 2021), the authors applied spatial transform to the YUV space to generate adversarial examples with higher superiority in image quality.

We summarized the adopted symbols in Table 1 to increase the readability.

**Table 1:** The notations used in this paper.

***x***	**clean example**	** C **	**the classifier**	***z*_*adv*_**	**the disturbed latent value**
*x*_*adv*_	adversarial example	L	loss function	δ	the noise
*y*	clean label	*F*	Pretrained Flow Model	*f*	the flow field
*t*	the target label	*z*	the latent value	N(·)	the four neighborhood

## 4. Methodology

In this section, we propose our attack method. First, we take an overview of our method. Next, we go over the detail of each part step by step. Finally, we discuss our objective function and summarize the whole process as Algorithm 1.

**Algorithm 1:** DualFlow attack.

**Input**: *X*_*tr*_: a batch of clean examples used for training; α: the learning rate; *T*: the maximal training iterations; *Q*: the maximal steps for attack; ξ: the flow budget; *X*_*te*_: a clean example used for test; C: the target model to be attacked.
**Output**: The adversarial example *x*_*adv*_ is used for attack.
**Parameter**: The flow model *F*_θ_.
1: Initialize the parameters of the flow model *F*_θ_;
2: for *i* = 1 to *T* **do**
3: Optimize *F*_θ_ according to Equation (6);
4: **if** Convergence reached **then**
5: break;
6: **end if**
7: **end for**
8: Obtain optimized *F*_θ_;
9: Compute the hidden representation of examples in *X*_*te*_ via $z=F^{-1}\left(x_{te}\right)$;
10: $z_{0}^{{}^{\prime }}=z$
11: Initialize the flow filed *f* with zeros;
12: **for** *i* = 1 to *Q* **do**
13: Optimize *f* via Equations (12) or 13;
14: Compute the adversarial example candidate $x_{i}^{{}^{\prime }}$ via Equation (11);
15: **if** Successfully attack C by $x_{i}^{{}^{\prime }}$ **then**
16: $x_{adv}=x_{i}^{{}^{\prime }}$
17: break.
18: **end if**
19: **end for**

### 4.1. The DualFlow framework

**Figure 2:**
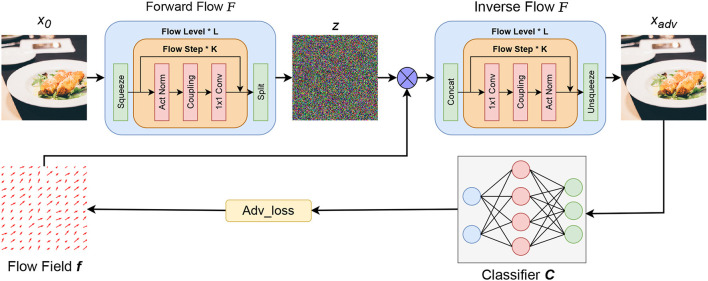
The framework of proposed DualFlow. *x* represent the image, among them, *x*_0_ is the benign image, *x*_*adv*_ is the corresponding adversarial counterpart; *z* is the hidden representation of the image; *F* is the well-trained Normalize Flow model and C is the pre-trained classifier; *f* is the flow field need to be optimized and ⊗ represents the spatial transform operation.

The proposed DualFlow attack framework can be divided into three parts, the first one is to map clean image *x* to its latent space *z* by the well-trained normalizing flow model. The second part is to optimize the flow field *f*, and apply it to the images' latent representation *z* and inverse the transformed *z* to generate its corresponding RGB space counterpart *x*_*t*_. Note that step 2 needs to be worked in an iterative manner to update the flow field *f* guided by the adv_loss until the adversarial candidate *x*_*t*_ can fool the target model. Finally, apply the optimized flow field *f* to the image's latent counterpart *z* and do the inverse operation of normalizing flow to obtain the adversarial image. The whole process is shown in Figure 2.

### 4.2. Normalizing flow model training

As introduced in Section 3.2., the training of the normalizing flow is to maximize the likelihood function on the training data with respect to the model parameters. Formally, assume that the collected dataset is denoted by *x*~*X*. The hidden representation follows the Gaussian distribution, i.e., z~N(0,1). The flow model is denoted by *F*, parameterized θ, which have *x* = *F*_θ_(*z*) and *z* = *F*^−1^(*x*). Then, the loss function to be minimized is expressed as:


(7) $$L(\theta ;z,x)=-\log p(x|z,\theta )=-\log p_{z}({\text{F}}_{\theta }^{-1}(x))-\log\left|det\frac{\partial {\text{F}}_{\theta }^{-1}(x)}{\partial x}\right|,$$

By optimizing the above objective, the learned distribution *p*(*x*|*z*, θ) characterizes the data distribution as expected.

In the training process, we use the Adam algorithm to optimize the model parameters; while the learning rate is set as 10^−4^, the momentum is set to 0.999, and the maximal iteration number is 100,000.

### 4.3. Generating adversarial examples with DualFlow

For a clean image *x*, to obtain its corresponding adversarial example *x*_*adv*_, we first calculate its corresponding latent space vector *z* by performing a forward flow process *via* *z* = *F*_θ_(*x*). Once the *z* is calculated, we can disturb it with the spatial transform techniques, the core is to optimize the flow filed vector *f*, which will be applied to *z* to get the transformed latent representation *z*_*st*_ according to *x*. In this paper, the flow filed vector *f* is directly optimized with the Adam optimizer iteratively. We will repeat the above process to optimize flow field *f* until *z*_*st*_ becomes an eligible adversarial latent value, that is, make the *z*_*st*_ becomes *z*_*adv*_. Finally, when the optimal flow filed *f* is calculated, we restore the transformed latent representation *z*_*adv*_ to the image space through the inverse operation of the normalizing flow model, that is, *x*_*adv*_ = *F*_θ_(*z*_*adv*_), to get its perturbed example *x*_*adv*_ in pixel level.

Moore specifically, the spatial transform techniques using a flow field matrix *f* = [2, *h, w*] to transform the original image *x* to *x*_*st*_ (Xiao et al., 2018). In this paper, we adopt the spatial transform from the pixel level to the latent space. Specifically, assume the latent representation of input *x* is *z* and its transformed counterpart *z*_*st*_, for the *i*-th value in *z*_*st*_ at the value location $\left(u_{st}^{i},v_{st}^{i}\right)$, we need to calculate the flow field matrix $f_{i}=\left(\Delta u^{i},\Delta v^{i}\right)$. So, the *i*-th value *z*^*i*^'s location in the transformed image can be indicated as:


(8) $$(u^{i},v^{i})=(u_{st}^{i}+\Delta u^{i},v_{st}^{i}+\Delta v^{i}).$$

To ensure the flow field *f* is differentiable, the bi-linear interpolation (Jaderberg et al., 2015) is used to obtain the four neighboring values surrounding the location $\left(u_{st}^{i}+\Delta u^{i},v_{st}^{i}+\Delta v^{i}\right)$ for the transformed latent value *z*_*st*_ as:


(9) $$z_{st}^{i}=\sum_{q\in \;N(u^{i},v^{i})}z^{q}(1-|u^{i}-u^{q}|)(1-|v^{i}-v^{q}|),$$

Where $N\left(u^{i},v^{i}\right)$ is the neighborhood, that is, the four positions (top-left, top-right, bottom-left, bottom-right) tightly surrounding the target value (*u*^*i*^, *v*^*i*^). In our adversarial attack settings, the calculated *z*_*st*_ is the final adversarial latent representation *z*_*adv*_. Once the *f* has been computed, we can obtain the *z*_*adv*_ by applying the calculated flow field *f* to the original *z*, which is given by:


(10) $$z_{adv}=\sum_{q\in N(u^{i},v^{i})}z^{q}(1-|u^{i}-u^{q}|)(1-|v^{i}-v^{q}|)),$$

and the adversarial examples *x*_*adv*_ can be obtained by:


(11) $$\begin{array}{l} x_{adv}=clip\left({\text{F}}^{-1}\left(z_{adv}\right),0,1\right), \end{array}$$

Where *clip*(·) is the clip operation to keep the generated value belonging to [0, 1].

### 4.4. Objective functions

Taking the attack success rate and visual invisibility of the generated adversarial examples into account, we divide the objective function into two parts, where one is the adversarial loss and the other is a constraint for the flow field. Unlike other flow field-based attack methods, which constrain the flow field by the flow loss proposed in Xiao et al. (2018), in our method, we use a dynamically updated flow field budget ξ (a small number, like 1*10^−3^) to regularize the flow field *f*. For adversarial attacks, the goal is making $C\left(x_{adv}\right)\neq y$. We give the objective function as follows:

for un-targeted attacks:


(12) $${ℒ}_{adv}(X,y,\text{f})=max[C{\left(X_{adv}\right)}_{y}-\underset{k\neq y}{max}C{\left(X_{adv}\right)}_{k},k],\;s.t.\|\text{f}\|\leq \xi .$$

for target attacks:


(13) $${ℒ}_{adv}(X,y,t,\text{f})=min[\underset{k=t}{max}C{\left(X_{adv}\right)}_{k}-C{\left(X_{adv}\right)}_{y},k],\;s.t.\|\text{f}\|\leq \xi .$$

The whole algorithm of LFFA is listed in Algorithm 1 for easy reproducing of our results, where lines 11-18 depict the core optimization process.

## 5. Experiments

In this section, we evaluate the proposed DualFlow on three benchmark image classification datasets. We first compare our proposed method with several baseline techniques concerned with Attack Success Rate (ASR) on clean models and robust models on three CV baseline datasets (CIFAR-10, CIFAR-100 and ImageNet). Then, we first provide a comparative experiment to the existing attack methods in image quality aspects with regard to LPIPS, DISTS, SCC, SSIM, VIPF and et al. Through these experimental results, we show the superiority of our method in attack ability, human inception and image quality.

### 5.1. Settings

#### Dataset

We verify the performance of our method on three benchmark datasets for computer vision task, named CIFAR-10^1^ (Krizhevsky and Hinton, 2009), CIFAR-100^1^ (Krizhevsky and Hinton, 2009) and ImageNet-1k^2^ (Deng et al., 2009). In detail, CIFAR-10 contains 50,000 training images and 10,000 testing images with the size of 3x32x32 from 10 classes; CIFAR-100 has 100 classes, including the same number of training and testing images as the CIFAR-10; ImageNet-1K has 1,000 categories, containing about 1.3M samples for training and 50,000 samples for validation. In particular, in this paper, we extend our attack on the whole images in testing datasets of CIFAR-10 and CIFAR-100, in terms of ImageNet-1k, we are using its subset datasets from ImageNet Adversarial Learning Challenge, which is commonly used in work related to adversarial attacks.

All the experiments are conducted on a GPU server with 4 * Tesla A100 40GB GPU, 2 * Xeon Glod 6112 CPU, and RAM 512GB.

#### Models

For CIFAR-10 and CIFAR-100, the pre-trained VGG-19 (Simonyan and Zisserman, 2015), ResNet-56 (He et al., 2016), MobileNet-V2 (Sandler et al., 2018) and ShuffleNet-V2 (Ma N. et al., 2018) are adopted, with top-1 classification accuracy 93.91, 94.37, 93.91, and 93.98% on CIFAR-10 and 73.87, 72.60, 71.13, and 75.49% on CIFAR-100, respectively, all the models' parameters are provided in the GitHub Repository^3^. For ImageNet, we use the PyTorch pre-trained clean model VGG-16, VGG-19 (Simonyan and Zisserman, 2015), ResNet-152 (He et al., 2016), MobileNet-V2 (Sandler et al., 2018) and DenseNet-121 (Huang et al., 2017), achieving 87.40, 89.00, 94.40, 87.80, and 91.60% classification accuracy rate on ImageNet, respectively. And in terms of robust models, they include Hendrycks2019Using (Hendrycks et al., 2019), Wu2020Adversarial (Wu et al., 2020), Chen2020Efficient (Chen et al., 2022) and Rice2020Overfitting (Rice et al., 2020) for CIFAR-10 and CIFAR-100, And Engstrom2019Robustness (Croce et al., 2021), Salman2020Do_R18 (Salman et al., 2020), Salman2020Do_R50 (Salman et al., 2020), and Wong2020Fast (Wong et al., 2020) for ImageNet. All the models we use are implemented in the robustbench toolbox^4^ (Croce et al., 2021) and the models' parameters are also provided in Croce et al. (2021). For all these models, we chose their *L*_*inf*_ version parameters due to most baselines being extended *L*_*inf*_ attacks in this paper.

#### Baselines

The baseline methods are FGSM (Goodfellow et al., 2015), MI-FGSM (Dong et al., 2018), TI-FGSM (Dong et al., 2019), Jitter (Schwinn et al., 2021), stAdv (Xiao et al., 2018), Chroma-Shift (Aydin et al., 2021), and GUAP (Zhang et al., 2020). The experimental results of those methods are reproduced by the Torchattacks toolkit^5^ and the code provided by the authors with default settings.

#### Metrics

Unlike the pixel-based attack methods, which only use *L*_*p*_ norm to evaluate the adversarial examples' perceptual similarity to its corresponding benign image. The adversarial examples generated by spatial transform always use other metrics referring to image quality. To be exact, in this paper, we follow the work in Aydin et al. (2021) using the following perceptual metrics to evaluate the adversarial examples generated by our method, including Learned Perceptual Image Patch Similarity (LPIPS) metric (Zhang et al., 2018) and Deep Image Structure and Texture Similarity (DISTS) index (Ding et al., 2022). LPIPS is a technique that measures the Euclidean distance of deep representations (i.e., VGG network Simonyan and Zisserman, 2015) calibrated by human perception. LPIPS has already been used on spatially transformed adversarial examples generating studies (Jordan et al., 2019; Laidlaw and Feizi, 2019; Aydin et al., 2021). DISTS is a method that combines texture similarity with structure similarity (i.e., feature maps) using deep networks with the optimization of human perception. We used the implementation of Ding et al. for both perceptual metrics (Ding et al., 2021). Moreover, we use other metrics like Spatial Correlation Coefficient (SCC) (Li, 2000), Structure Similarity Index Measure (SSIM) and Pixel Based Visual Information Fidelity (VIFP) (Sheikh and Bovik, 2004) to assess the generated images' qualities. SCC reflects the indirect correlation based on the spatial contiguity between any two geographical entities. SSIM is used to assess the generated images' qualities concerning luminance, contrast and structure. VIFP is used to assess the adversarial examples' image quality. The primary toolkits we used in the experiments of this part are IQA_pytorch^6^ and sewar^7^.

### 5.2. Quantitative comparison with the existing attacks

In this subsection, we will evaluate the proposed DualFlow and the baselines FGSM, MI-FGSM, TI-FGSM (Dong et al., 2019), Jitter, stAdv, Chroma-shift and GUAP in attack success rate on CIFAR-10, CIFAR-100 and the whole ImageNet dataset. We set the noise budget as ϵ = 0.031 for all *L*_*inf*_-based attacks baseline methods. The other attack methods, such as stAdv and Chroma-shift, follow their default settings in the code provided by the authors.

Tables 2–4 show the ASR of DualFlow and the baselines on CIFAR-10, CIFAR-100 and ImageNet, respectively. As the results illustrated, DualFlow can perform better in most situations on the three benchmark datasets. Take the attack results on ImageNet as an example, refer to Table 3. The BIM, MI-FGSM, TI-FGSM, Jitter, stAdv, Chroma-shift and GUAP can achieve 91.954, 98.556, 93.94, 95.172, 97.356, 98.678, and 94.606% average attack success rate on ImageNet dataset, respectively, vice versa, our DualFlow can achieve 99.364% average attack success rate. On the other two benchmark datasets, CIFAR-10 and CIFAR-100, the DualFlow also can get a better average attack performance. To further explore the attack performance of the proposed DualFlow, we also extend the targeted attack on ImageNet, and the results are presented in Table 4. The empirical results show that DualFlow can generate more powerful adversarial examples and obtain a superior attack success rate in most cases. It can get an ASR range from 94.12 to 99.52% on five benchmark DL models, but the most competitive baseline MI-FGSM can achieve an ASR of 83.90 to 99.34%. It is indicated that the proposed method is more threatening to DNNs and meaningful for exploring the existing DNNs' vulnerability and guiding the new DNNs' design.

**Table 2:** Experimental results on attack success rate (ASR) of un-targeted attack of CIFAR-10 and CIFAR-100.

	**CIFAR-10**				**CIFAR-100**			
	**VGG19**	**ResNet56**	**MobileNetV2**	**ShuffleNetV2**	**VGG19**	**ResNet56**	**MobileNetV2**	**ShuffleNetV2**
FGSM	55.28	65.58	71.46	54.85	75.42	91.23	90.40	85.72
MI-FGSM	76.43	93.11	94.12	78.47	87.69	99.78	99.47	93.68
TI-FGSM	59.63	71.03	80.01	76.10	83.43	97.46	93.92	92.77
Jitter	83.70	94.87	**96.92**	86.25	98.31	**100.00**	**99.76**	94.63
stAdv	86.04	63.77	69.43	66.11	97.66	93.26	93.55	95.61
Chroma-shift	84.87	68.36	73.57	64.58	98.84	98.37	96.39	96.86
GUAP	82.55	89.34	87.61	87.02	92.26	94.59	96.89	92.20
DualFlow	**97.07**	**95.31**	93.65	**96.19**	**99.32**	99.02	98.83	**97.36**

The victim models are VGG19, ResNet56, MobileNetV2 and ShuffleNetV2, respectively, pre-trained by a GitHub Repository, named pytorch-cifar-models. Note that for the FGSM-based baselines, we synthesize their adversarial examples under *L*_*inf*_-norm=0.031 limitation; the others are not subject to the *L*_*inf*_-norm restrictions. Bold values indicates the best result.

**Table 3:** Experimental results on attack success rate (ASR) of un-targeted attack of ImageNet.

	**GSM**	**MI-FGSM**	**TI-FGSM**	**Jitter**	**stAdv**	**Chroma-shift**	**GUAP**	**DualFlow**
VGG16	93.56	98.64	97.16	95.27	97.62	98.62	97.73	**99.37**
VGG19	95.31	99.42	96.34	91.76	98.74	98.98	96.10	**99.43**
ResNet152	84	96.82	85.17	94.28	97.46	97.79	88.90	**98.63**
MobileNetV2	91.92	98.29	91.47	94.99	96.13	99.35	97.60	**99.61**
DenseNet121	94.98	99.61	99.56	99.56	96.83	98.65	92.70	**99.78**

The victim models are VGG19, ResNet152, MobileNetV2 and DenseNet121, respectively, which are pre-trained by PyTorch. Note that for the FGSM-based baselines, we synthesize their adversarial examples under *L*_*inf*_-norm=0.031 limitation; the others are not subject to the *L*_*inf*_-norm restrictions. Bold values indicates the best result.

**Table 4:** Experimental results on the attack success rate of targeted attack on dataset ImageNet.

**Methods**	**FGSM**	**MI-FGSM**	**TI-FGSM**	**Jitter**	**stAdv**	**Chroma-Shift**	**DualFlow**
VGG16	80.78	73.11	96.34	67.51	54.74	65.10	**96.67**
VGG19	60.59	49.36	83.90	46.50	53.23	55.39	**98.85**
ResNet152	80.22	73.93	**94.72**	70.45	65.87	69.60	94.12
MobileNetV2	72.70	63.94	92.38	60.86	70.63	76.00	**99.52**
DenseNet121	78.06	74.56	**99.34**	63.86	75.94	80.79	99.06

The baselines are FGSM, MI-FGSM, TI-FGSM, Jitter, stAdv, Chroma-shift and DualFlow. Note that for the FGSM-based baselines, we synthesize their adversarial examples under *L*_*inf*_-norm=0.031 limitation; the others are not subject to the restrictions. Bold values indicates the best result.

### 5.3. Attack on defense models

Next, we investigate the performance of the proposed method in attacking robust image classifiers. Thus we select some of the most recent defense techniques that are from the robustbench toolbox as follows, for CIFAR-10 and CIFAR-100 are Hendrycks2019Using (Hendrycks et al., 2019), Wu2020Adversarial (Wu et al., 2020), Chen2020Efficient (Chen et al., 2022) and Rice2020Overfitting (Rice et al., 2020); for ImageNet are Engstrom2019Robustness (Croce et al., 2021), Salman2020Do_R18 (Salman et al., 2020), Salman2020Do_R50 (Salman et al., 2020) and Wong2020Fast (Wong et al., 2020). We compare our proposed method with the baseline methods.

Following the results shown in Table 5, we derive that DualFlow exhibits the best performance of all the baseline methods in terms of the attack success rate in most cases. The attack success rate of the baseline method stAdv and Chroma-Shift range from 95.41 to 99.12% and 17.22% from 74.80 in ImageNet, respectively. However, the DualFlow can obtain a higher performance range from 97.50 to 100%. It demonstrates the superiority of our method when attacking robust models.

**Table 5:** Experimental results on the attack success rate of un-targeted attack on CIFAR-10, CIFAR-100 and ImageNet dataset to robust models.

		**FGSM**	**MIFGSM**	**TIFGSM**	**Jitter**	**stAdv**	**Chroma-shift**	**DualFlow**
CIFAR-10	Hendrycks2019Using	27.06	16.90	18.54	32.67	99.12	20.70	**100**
	Wu2020Adversarial	25.63	16.28	19.10	31.02	99.12	18.36	**100**
	Chen2020Efficient	28.59	18.93	20.94	35.59	99.02	24.90	**100**
	Rice2020Overfitting	27.38	16.87	16.92	33.02	98.93	25.98	**100**
CIFAR-100	Hendrycks2019Using	37.67	25.57	28.88	48.89	95.41	35.16	**100**
	Wu2020Adversarial	40.13	27.06	30.71	50.13	97.66	30.86	**100**
	Chen2020Efficient	42.24	30.51	34.24	54.66	97.75	34.57	**100**
	Rice2020Overfitting	52.55	38.92	46.63	62.66	97.75	34.67	**100**
ImageNet	Engstrom2019Robustness	62.92	51.03	65.50	83.85	95.41	22.61	**97.50**
		Salman2020Do_R18	65.61	51.82	62.44	82.09	97.66	42.16	**100**
		Salman2020Do_R50	57.58	44.99	55.66	76.48	97.75	17.22	**99.19**
		Wong2020Fast	61.24	50.08	70.02	82.30	**97.75**	74.80	97.5

Bold values indicates the best result.

### 5.4. Evaluation of human perceptual and image quality

Unlike the noise-adding attack methods, which usually use *L*_*p*_ norm to evaluate the victim examples' perceptual similarity to its corresponding benign image. The adversarial examples generated by noise-beyond ways always use other metrics referring to image quality. To be exact, we follow the work in Aydin et al. (2021) using the following perceptual metrics to evaluate the adversarial examples generated by baseline methods and the proposed method, including Learned Perceptual Image Patch Similarity (LPIPS) metric (Zhang et al., 2018) and Deep Image Structure and Texture Similarity (DISTS) index (Ding et al., 2022). In addition, *L*_*inf*_-norm, Spatial Correlation Coefficient (SCC) (Li, 2000), Structure Similarity Index Measure (SSIM) (Wang et al., 2004), and Pixel Based Visual Information Fidelity (VIFP) (Sheikh and Bovik, 2004) are also involved in evaluating the difference between the generated adversarial examples and their benign counterparts and the quality of the generated adversarial examples.

The generated images' quality results can be seen in Table 6, which indicated that the proposed method has the lowest LPIPS, DISTS perceptual loss and *L*_*inf*_ (the lower is better) are 0.0188, 0.0324 and 0.1642, respectively, on VGG-19 model; and has the highest SCC, SSIM and VIFP (the higher is better), achieving 0.9452, 0.7876 and 0.8192, respectively, on VGG-19 model. All the empirical data are obtained on the ImageNet dataset. The results show that the proposed method is superior to the existing attack methods.

**Table 6:** Perceptual distances were calculated on fooled examples by FGSM, MI-FGSM, TI-FGSM, Jitter, stAdv, Chroma-shift, GUAP, and the proposed DualFlow on ImageNet.

	**VGG19**						**ResNet152**					
	**LPIPS**	**DISTS**	*L*_*inf*_	**SCC**	**SSIM**	**VIFP**	**LPIPS**	**DISTS**	*L*_*inf*_	**SCC**	**SSIM**	**VIFP**
FGSM	0.3036	0.1916	–	0.5572	0.8273	0.4705	0.2688	0.1679	–	0.5796	0.8348	0.4753
MI-FGSM	0.1962	0.1444	–	0.7135	0.9474	0.6575	0.1589	0.1078	–	0.7180	0.9466	0.6597
TI-FGSM	0.2179	0.1849	–	0.8153	0.9199	0.5576	0.1684	0.1451	–	0.8216	0.9330	0.5943
Jitter	0.2461	0.1617	–	0.6342	0.9076	0.5864	0.2001	0.1305	–	0.6480	0.9107	0.5792
stAdv	0.0581	0.0757	0.2420	0.8954	0.9873	0.7290	0.0490	0.0690	0.2420	0.8954	0.9873	0.7290
Chroma-shift	0.0231	0.5943	0.0275	0.9142	0.9834	0.8079	0.0.0203	0.0246	0.0.2250	0.9126	0.0.9848	0.0.8027
GUAP	0.4349	0.2838	0.4984	0.2768	0.7630	0.2955	0.4205	0.2501	0.6443	0.2289	0.7274	0.2674
DualFlow	**0.0188**	**0.0324**	**0.1642**	**0.9451**	**0.9876**	**0.8192**	**0.0169**	**0.0312**	**0.1550**	**0.9451**	**0.9876**	**0.8192**

Note that for the FGSM-based baselines, we synthesize their adversarial examples under *L*_*inf*_-norm=0.031 limitation; the others are not subject to the restrictions. Bold values indicates the best result.

**Figure 3:**
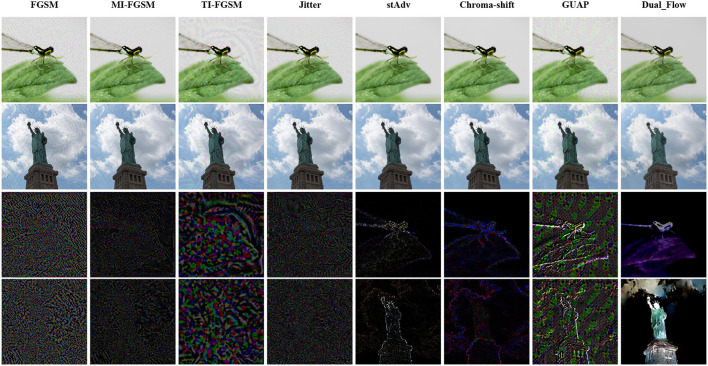
Adversarial examples and their corresponding perturbations. The first two columns are the adversarial examples, and the followings are the adversarial noise of FGSM, MI-FGSM, TI-FGSM, Jitter, stAdv, Chroma-shift, GUAP and our method, respectively.

To visualize the difference between the adversarial examples generated by our method and the baselines, we also draw the adversarial perturbation generated on NIPS2107 by FGSM, MI-FGSM, TI-FGSM, Jitter stAdv, Chroma-shift, GUAP and the proposed method in Figure 3, the target model is pre-trained VGG-19. The first two columns is the adversarial examples and the following are the adversarial noises of FGSM, MI-FGSM, TI-FGSM, Jitter stAdv, Chroma-shift, GUAP and our method, respectively. Noted that, for better observation, we magnified the noise by a factor of 10. From Figure 3, we can clearly observe that stAdv and Chroma-Shift distort the whole image. In contrast, the adversarial examples generated by our method are focused on the salient region and its noise is milder, and they are similar to the original clean counterparts and are more imperceptible to human eyes. These simulations of the proposed method take place under diverse aspects and the outcome verified the betterment of the presented method over the compared baselines.

### 5.5. Detectability

Adversarial examples can be viewed as data outside the clean data distribution, so the defender can easily check whether each input is an adversarial example. Therefore, generating adversarial examples with high concealment means that they have the same or similar distribution as the original data (Ma X. et al., 2018; Dolatabadi et al., 2020). To verify whether the carefully crafted examples satisfy this rule, we follow (Dolatabadi et al., 2020) and select LID (Ma X. et al., 2018), Mahalanobis (Lee et al., 2018), and Res-Flow (Zisselman and Tamar, 2020) adversarial attack detectors to evaluate the performance of the adversarial examples crafted by DualFlow. For comparison, we choose FGSM (Goodfellow et al., 2015), MI-FGSM (Dong et al., 2018), stAdv (Xiao et al., 2018), and Chroma-Shift (Aydin et al., 2021) as baseline methods. The test results are shown in the Table 7, including the area under the receiver operating characteristic curve (AUROC) and detection accuracy. Table 7, we can find that these adversarial detectors struggle to detect malicious examples constructed with DualFlow, compared to the baseline in all cases. Empirical results precisely demonstrate the superiority of our method, which generates adversarial examples closer to the distribution of original clean images than other methods, and the optimized adversarial perturbations have better hiding ability. The classifier is ResNet-34, and the code used in this experiment is modified from deep_Mahalanobis_detector^8^ and Residual-Flow^9^, respectively.

**Table 7:** The detect results of DualFlow and the baselines on CIFAR-10 and CIFAR-100, Where the Chroma represent the Chroma-Shift.

**Datasets**	**Methods**	**AUROC (%)** ↑					**Detection Acc. (%)** ↑				
**FGSM**	**MI-FGSM**	**stAdv**	**Chroma**	**DualFlow**	**FGSM**	**MI-FGSM**	**stAdv**	**Chroma**	**DualFlow**
CIFAR-10	LID	99.67	95.36	82.13	70.61	**52.23**	99.73	90.42	78.95	65.42	**58.42**
	Mahalanobis	96.54	98.54	85.64	75.61	**58.49**	90.42	97.26	79.67	76.13	**64.23**
	Res-Flow	94.47	97.59	78.96	72.37	**64.95**	88.56	91.54	76.38	73.64	**59.78**
CIFAR-100	LID	97.86	91.67	75.85	73.84	**62.37**	93.34	82.6	76.71	69.57	**57.78**
	Mahalanobis	99.61	97.64	76.17	72.32	**65.48**	98.62	92.49	80.65	71.48	**63.15**
	Res-Flow	99.07	99.76	78.53	78.56	**65.74**	95.92	96.99	83.43	69.72	**62.94**

↑ means that the larger the value, the better the detection method. Bold values indicates the best result.

## 6. Conclusions

In this paper, we propose a novel framework named Dual-Flow for generating imperceptible adversarial examples with strong attack ability. It aims to perturb images by disturbing their latent representation space rather than adding noise to the clean image at the pixel level. Combining the normalizing flow and the spatial transform techniques, DualFlow can attack images' latent representations by changing the position of each value in the latent vector to craft adversarial examples. Besides, the empirical results of defense models show that DualFlow has stronger attack capability than noise-adding-based methods, which is meaningful for exploring the DNN's vulnerability sufficiently. Therefore, developing a more effective method to generate invisible, both for human eyes and the machine, is fascinating. Extensive experiments show that the adversarial examples obtained by DualFlow have superiority in imperceptibility and attack ability compared with the existing methods.

## Data availability statement

Publicly available datasets were analyzed in this study. This data can be found at: CIFAR-10 and CIFAR-100, http://www.cs.toronto.edu/~kriz/cifar.html; ImageNet, https://image-net.org/.

## Author contributions

YW and JinZ performed computer simulations. DH analyzed the data. RL and JinhZ wrote the original draft. RL and XJ revised and edited the manuscript. WZ polished the manuscript. All authors confirmed the submitted version.

## Footnotes

^1^ http://www.cs.toronto.edu/~kriz/cifar.html

^2^ https://image-net.org/

^3^ https://github.com/chenyaofo/pytorch-cifar-models

^4^ https://github.com/RobustBench/robustbench

^5^ https://github.com/Harry24k/adversarial-attacks-pytorch

^6^ https://www.cnpython.com/pypi/iqa-pytorch

^7^ https://github.com/andrewekhalel/sewar

^8^ https://github.com/pokaxpoka/deep_Mahalanobis_detector

^9^ https://github.com/EvZissel/Residual-Flow
